# Vascular Events, Vascular Disease and Vascular Risk Factors—Strongly Intertwined with COVID-19

**DOI:** 10.1007/s11940-020-00648-y

**Published:** 2020-10-08

**Authors:** Adrian Scutelnic, Mirjam R. Heldner

**Affiliations:** Department of Neurology, Inselspital, University Hospital and University of Bern, Bern, Switzerland

**Keywords:** COVID-19, Ischaemic stroke, Vascular events, Vascular risk factors, Prevention

## Abstract

**Purpose of review:**

To elucidate the intertwining of vascular events, vascular disease and vascular risk factors and COVID-19.

**Recent findings:**

Strokes are a leading cause of disability and death worldwide. Vascular risk factors are important drivers of strokes. There are unmodifiable vascular risk factors such as age and ethnicity and modifiable vascular risk factors. According to the INTERSTROKE study, the 10 most frequent modifiable vascular risk factors are arterial hypertension, physical inactivity, overweight, dyslipidaemia, smoking, unhealthy diet, cardiac pathologies, diabetes mellitus, stress/depression and overconsumption of alcohol. Also, infection and inflammation have been shown to increase the risk of stroke. There is high-quality evidence for the clinical benefits of optimal primary and secondary stroke prevention. The COVID-19 pandemic brought a new perspective to this field. Vascular events, vascular disease and vascular risk factors—and COVID-19—are strongly intertwined. An increased risk of vascular events—by multifactorial mechanisms—has been observed in COVID-19 patients. Also, a higher rate of infection with COVID-19, severe COVID-19 and bad outcome has been demonstrated in patients with pre-existing vascular disease and vascular risk factors.

**Summary:**

At present, we suggest that regular interactions between healthcare professionals and patients should include education on COVID-19 and on primary and secondary vascular prevention in order to reduce the burden of disease in our ageing populations.

## COVID-19—vascular events, vascular disease and vascular risk factors

The infection, caused by a novel coronavirus, the so-called severe acute respiratory syndrome (SARS)-coronavirus (CoV)-2, has been named COVID-19 (coronavirus disease 2019) by the WHO (https://www.who.int/). The clinical presentation of COVID-19 varies. Most people experience an asymptomatic infection or mild symptoms with no or mild pneumonia. In some people, severe symptoms are observed, and in few people, critical symptoms occur, with respiratory failure, septic shock and/or multiorgan dysfunction or failure, potentially leading to death. The fatality rate has been shown to vary widely depending on, e.g., age, sex, co-morbidities, vascular disease and vascular risk factors [1–11]. Without any interventions such as social distancing and hygiene measures, a person infected with SARS-CoV-2 is likely to transmit COVID-19 to at least two persons [1]. The causality between vascular events, vascular disease and vascular risk factors—and COVID-19—seems to be interconnected and multidirectional [12].

## Search strategy and study selection

We carried out a literature search on PubMed, WHO’s homepage, SCOPUS, EuropePMC and Cochrane Central Database with the following search terms (1) “COVID-19” OR “SARS-CoV-2” AND “characteristics,” (2) “COVID-19” OR “SARS-CoV-2” AND “cerebrovascular,” (3) “COVID-19” OR “SARS-CoV-2” AND “cardiovascular,” (4) “COVID-19” OR “SARS-CoV-2” AND “stroke,” (5)“COVID-19” OR “SARS-CoV-2” AND “myocardial infarction,” (6)“COVID-19” OR “SARS-CoV-2” AND “co-morbidities” and (7) “COVID-19” OR “SARS-CoV-2” AND all risk factors according to the INTERSTROKE study. The authors independently performed a search and screening for relevant articles through title and abstract. The literature search was finalized on July 18, 2020.

## COVID-19 and risk of vascular events

In Middle-East Respiratory Syndrome (MERS) and SARS local occlusion of small- and mid-sized vessels have been described, having caused venous thrombosis, embolism and ischaemic as well as haemorrhagic stroke [13]. Vascular events also have been identified in patients infected by COVID-19 [13–24]. Here, we focus on vascular events in COVID-19 patients affecting the arteries or the brain-supplying vessels only. In patients with pre-existing vascular disease and vascular risk factors, COVID-19 likely advances the foreseeable vascular event to an earlier time point, but also previously healthy individuals with COVID-19 can be affected by vascular events [17].

A retrospective cohort study showed higher rates of TIA, ischaemic and haemorrhagic stroke at admission in patients with vs. without COVID-19 (*n* = 173) (76.8% vs. 58.1%; *p* = 0.018) [16].

In an observational study (*n* = 388 COVID-19 patients, 16% admitted to ICU), ischaemic strokes were diagnosed in 2.5% of all patients within a median duration of hospitalization of 10 (7–15) days. Two-thirds of those events were the reason for admission. Acute coronary syndrome/myocardial infarction was diagnosed in 1.1% of all patients; in 75%, it was the reason for admission [22].

In a retrospective observational case series (*n* = 214, 41.1% with severe COVID-19), a proportion of around 5% of hospitalized COVID-19 patients also had a stroke. Higher rates of stroke were seen in patients with severe vs. non-severe COVID-19 (5.7% vs. 0.8%; *p* = 0.03). The same group later published another report (*n* = 221), in which they identified 85% of strokes as ischaemic, one intracranial haemorrhage and one cerebral venous thrombosis. Thirty-eight percent of these stroke patients died. Furthermore, the authors showed that besides those patients with severe COVID-19, older patients and those with higher vascular risk profile or C-reactive protein or D-dimer levels were at significantly higher risk of stroke [15, 23].

A retrospective cohort study (*n* = 3556 COVID-19 patients) identified 32 patients (0.9%) with radiologically proven ischaemic stroke, 75% of which had severe disease or died. Stroke was the reason for admission in 43.8% of patients, and 15.6% had no COVID-19 symptoms prior to ischaemic stroke [18]. This study showed cryptogenic vs. other stroke aetiology to be more frequent in COVID-19 patients if compared with contemporary controls (65.6% vs. 30.4%; *p* = 0.003) and with controls from an earlier time period (65.6% vs. 25%; *p* < 0.001) [18].

**Fig. 1:**
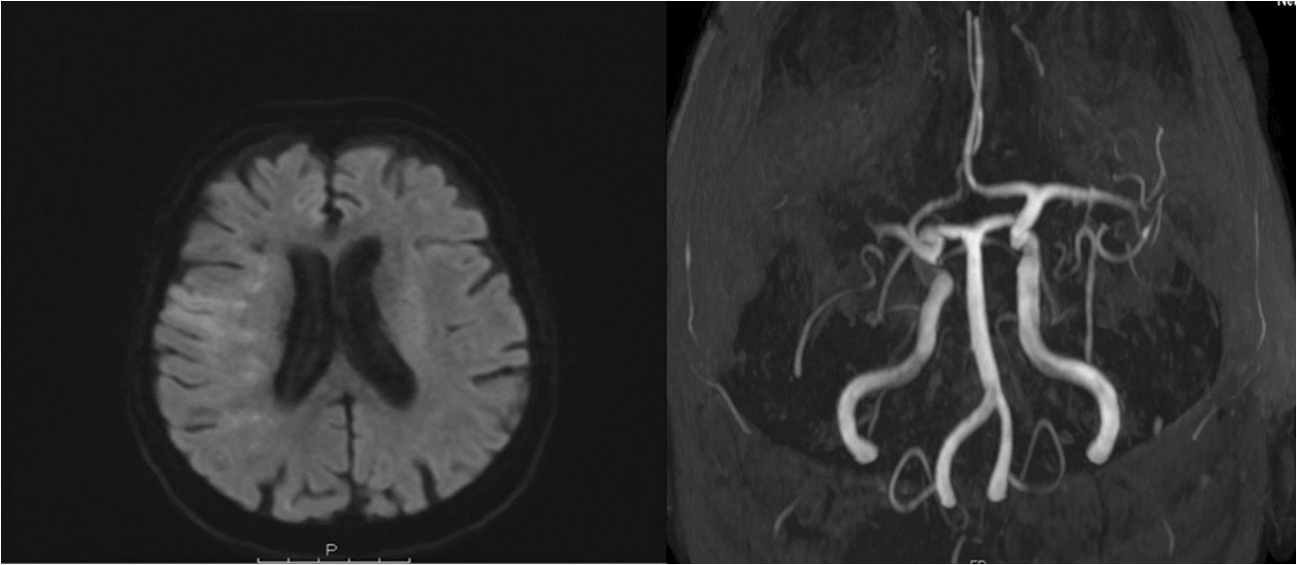
Patient with ischaemic stroke because of large vessel occlusion. An 86-year-old female patient was brought by ambulance to our emergency department because of left-sided hemiparesis, forced eye deviation towards the right and confusion. She had a history of atrial fibrillation, coronary heart disease and diabetes mellitus type 2. The initial evaluation revealed an NIHSS score of 17. Because her levels of anti-Xa were elevated due to recent intake of rivaroxaban, an endovascular therapy with thrombectomy (successful reperfusion: TICI 2b) without intravenous thrombolysis was performed. One day after intervention, the clinical assessment showed a residual NIHSS score of 8 because of moderate left-sided hemiparesis, left-sided facial droop and dysarthria.

In a case series, acute ischaemic stroke because of large vessel occlusion (Fig. 1) was reported as the presenting feature of COVID-19 in five patients younger than 50 years old without otherwise severe COVID-19. One patient had a history of mild stroke and diabetes mellitus, one an undiagnosed diabetes mellitus, one dyslipidaemia and arterial hypertension and two patients no vascular risk factors [24•]. 

A meta-analysis estimated the prevalence of ischaemic stroke in COVID-19 patients to be 1.6% (95% CI 0.8–2.5%), although there was a substantial heterogeneity between studies (*I*^2^ = 47%) [25].

The mechanisms increasing the risk of vascular events in COVID-19 patients are multifactorial [12, 15, 22, 26]. 

Studies have found pathological laboratory test results in COVID-19 patients, especially in those with severe COVID-19, such as elevated leukocyte counts, reduced lymphocyte counts and elevated levels of C-reactive protein, D-dimer levels, ferritin and lactate dehydrogenase, indicating hypercoagulability that could potentially provoke vessel occlusion and embolism [15, 26].

Pro-inflammatory cytokines are known to trigger hypercoagulability. However, in a retrospective cohort study, elevated levels of the cytokine interleukin-6 were discrepant with elevated D-dimer levels in non-survivors. Interleukin-6 levels appeared to increase at 2 weeks after COVID-19 onset, whereas D-dimer levels were already 10-fold increased by that time. This observation suggests that very high D-dimer levels observed in some COVID-19 patients are not only secondary to systemic inflammation, but likely reflect true thrombotic disease, possibly induced by cellular activation triggered by SARS-CoV-2 [14].

Antiphospholipid antibodies can be a cause of hypercoagulability with subsequent vessel occlusions. Elevated titres of antiphospholipid antibodies have been detected in some patients with COVID-19 and ischaemic stroke or acute limb ischaemia. However, causality remains uncertain [26].

SARS-CoV-2 may increase the risk of stroke by infection of the vascular endothelial cells causing endotheliitis. The resulting damage of the vasculature may lead to vascular events, including venous thrombosis, embolism and ischaemic or haemorrhagic stroke [27••].

Moreover, an acute COVID-19 cardiovascular syndrome (ACovCS) has been described [12]. This may be caused by viral myocarditis, systemic inflammation, stress-related cardiomyopathy, or microvascular thrombosis. Myocardial injury, heart failure and/or cardiac arrhythmia may occur leading to macrovascular thrombus formation and potentially to embolism or less frequently to hypoperfusion—the latter in patients with pre-existing high-grade vessel stenoses or occlusions of the brain-supplying arteries. Also, venous thrombi might be dislodged and cause paradoxical embolism to the brain.

To conclude, vascular events can occur in COVID-19 patients by several mechanisms. These events may be clinically silent, occur as the presenting symptom, or during the disease course and potentially lead to disability and death. While vascular events in COVID-19 patients have been detected in healthy persons, the risk is higher with severe COVID-19, and also in patients with pre-existing vascular disease and vascular risk factors.

## COVID-19—vascular disease and vascular risk factors

A higher rate of infection with COVID-19, severe COVID-19, and worse outcome has been demonstrated in patients with pre-existing vascular disease and risk factors, compared with young and healthy persons [1, 6, 8–11, 28, 29].

In a meta-analysis (*n* = 1576, 280 with severe COVID-19), the most prevalent co-morbidities in COVID-19 patients turned out to be arterial hypertension (21.1%, 95% CI = 13.0–27.2%), diabetes mellitus (9.7%, 95% CI = 7.2–12.2%), cardiovascular disease (8.4%, 95% CI = 3.8–13.8%) and respiratory disease (1.5%, 95% CI = 0.9–2.1%) [28]. Other studies showed similar results [1, 5–11].

A systematic review, meta-analysis/meta-regression (16 studies, *n* = 4448 COVID-19 patients, 19.8% died, 56.7% with severe COVID-19) revealed that besides pre-existing cardiovascular disease, cerebrovascular disease was also associated with poor outcome (RR 2.04, 95% CI = 1.43–2.91; *p* < 0.001), mortality (RR 2.38, 95% CI = 1.92–2.96; *p* < 0.001) and severe COVID-19 (RR 1.88, 95% CI = 1.0–3.51; *p* = 0.05). The association was not influenced by gender, age, arterial hypertension, diabetes mellitus and respiratory co-morbidities [29•].

A single-centre retrospective cohort study (*n* = 1875 COVID-19 patients, 50 with previous stroke of which 90% were ischaemic) showed that previous vs. no previous stroke was more likely associated with acute respiratory distress syndrome (ARDS) (32% vs. 18.9%; *p* = 0.028), with non-invasive mechanical ventilation (30% vs. 17.5%; *p* = 0.037) and with death (14.0% vs. 8.3%; *p* = 0.019) [30].

In a descriptive study (*n* = 1591 ICU-treated COVID-19 patients), pre-existing vascular disease was associated with severe COVID-19 and in 21% of patients it was the second most frequent co-morbidity after arterial hypertension (49%) [11].

In another descriptive study of the clinical characteristics of 138 COVID-19 patients, ICU-treated patients (*n* = 36, 26.1%) were more likely to suffer from co-morbidities such as high blood pressure (58.3% vs. 21.6% in the non-ICU group; *p* < 0.001), diabetes mellitus (22.2% vs. 5.9%; *p* = 0.009) and vascular disease (25% vs. 10.8%; *p* = 0.04). Also, the ICU group was significantly older (median age 66 vs. 51 years; *p* < 0.001) [1].

The elderly are more likely to suffer from co-morbidities and from additional vascular risk factors besides age [1]. Furthermore, the immune system declines with age. In the elderly, there is lymphopenia, decreased regulatory T cell function and clearance of apoptotic cells by macrophages as well as elevated levels of pro-inflammatory cytokines interleukin-1beta, interleukin-6, interleukin-18 and TNF-alfa [31]. This may contribute to the burden of COVID-19 in the elderly.

In addition, lockdown, social distancing and economic downturn are potentially changing lifestyles, dietary and smoking habits, reducing physical activity and increasing obesity and mental health problems. These changes impact the vascular risk profile in some people [32, 33].

To conclude, optimal primary and secondary vascular prevention might reduce the burden of COVID-19. Below, we review specific and potentially modifiable vascular risk factors with respect to COVID-19 and stroke.

## Arterial hypertension

In a meta-analysis (*n* = 1576, 280 with severe COVID-19), arterial hypertension was the most prevalent vascular risk factor in COVID-19 patients (21.1%, 95% CI = 13.0–27.2%) [28]. Also, outcome of COVID-19 has been shown to be worse in patients with arterial hypertension [34]. While confounding factors such as age and co-morbidities may explain these findings, a role has been postulated for angiotensin-converting enzyme 2 (ACE2) which is the binding site and entry receptor of SARS-CoV-2 [35••]. In patients with arterial hypertension, ACE2 levels are increased in the blood, and this results in enhanced angiotensin II effects. SARS-CoV-2 leads to a partial decrease in ACE2 function, which also leads to enhanced angiotensin II effects [36••]. Angiotensin II triggers inflammation, cell proliferation, hypertrophy, fibrosis and tissue remodeling through angiotensin 1 (AT1) receptor (so-called ACE/angiotensin II/AT1 receptor axis) [37].

Angiotensin-converting enzyme inhibitors (ACEIs) and angiotensin receptor blockers (ARBs), which are frequently prescribed as antihypertensive drugs, increase the expression of ACE2 in tissue. They have been discussed to have a potentially adverse, protective and/or biphasic effect on COVID-19 [36••]. Increased expression of ACE2 in tissue may antagonize some detrimental effects of SARS-CoV-2. In contrast, ACEIs/ARBs might negatively impact infection risk and outcome by increasing binding sites. A biphasic effect might be explained by the different phases of acute infection, which have been shown in COVID-19 [36••].

Some studies found a better COVID-19 outcome associated with ACEIs/ARBs [38–41], while others did not [42–44]. One retrospective descriptive study (*n* = 113 COVID-19 patients, 74 on ACEIs/ARBs) found increased mortality in patients on ACEIs/ARBs (OR 3.66, 95% CI 1.11–18.18, *p* = 0.032) [45]. Patients on ACEIs/ARBs were older and more likely to have coronary artery disease. Also, only patients with moderate or severe COVID-19 were included. The authors acknowledged the possibility of unrecognized confounders and the low number of patients as study limitations.

At present, current guidelines do not recommend to prophylactically prescribe ACEIs/ARBs to decrease COVID-19 infection risk and to improve outcome of COVID-19, and they do not recommend to switch an established treatment for these antihypertensive drugs in COVID-19 patients [38]. Sudden cessation of antihypertensive drugs increases the risk of vascular events, and newly initiated antihypertensive drugs may be less effective and/or tolerated.

## Physical inactivity

Physical activity has a positive impact on weight, blood pressure and glucose, dyslipidaemia, cigarette craving, anxiety/depression and systemic inflammation, which all increase vascular risk [46]. Physical activity also improves immune response to vaccines and decreases duration and severity of viral illness mainly through increased levels of antiinflammatory cytokines (e.g., interleukin-10, interleukin-1 receptor antagonist) and through downregulation of expression/activation of toll-like receptors (TLRs) [47].

Social distancing might facilitate personal instead of public transportation and thus increase physical activity. A cross-sectional analysis in one study found a doubling of the average daily use of a Public Bicycle Sharing System in 2020 compared with the same period in 2019 [48]. However, social distancing and lockdown policies, economic downturn and fear of contracting COVID-19 have also been found to negatively impact physical activity [49].

A descriptive study (*n* = 455,404, 187 countries) using data from a smartphone step-counting app found a 5.5% decrease of steps within 10 days and a 27.3% decrease within 30 days after declaration of the COVID-19 pandemic [50].

An electronic survey (*n* = 2524) found significantly decreased levels of physical activity during COVID-19 lockdown compared with before lockdown in all age groups (mean 2429 vs. 1577 metabolic equivalent task min/week; *p* < 0.0001) [51].

A study including 109 children with congenital heart disease showed a decrease of steps by 21–24% around March 11, when the WHO declared the COVID-19 outbreak to be a pandemic [52].

One survey (*n* = 9456) showed that a decrease of physical activity during lockdown was associated with obesity (OR 1.21, 95% CI = 1.02–1.41), arterial hypertension (OR 1.52, 95% CI = 1.33–1.71), pulmonary disease (OR 1.31, 95% CI = 1.13–1.49), depression (OR 2.02, 95% CI = 1.82–2.22) and disability (OR 2.34, 95% CI = 1.99–2.69) [33].

A prospective cohort study (*n* = 387,109 of whom 760 had COVID-19 between March/April 2020) found physical inactivity (quantified using the International Physical Activity Questionnaire) to be a risk factor for COVID-19 (OR 1.32, 95% CI 1.10–1.58) [53•].

Physical activity might help to prevent infection and severity of COVID-19, severity by attenuating the cytokine storm syndrome that occurs in some patients [47].

There are options for at-home physical activity that should be encouraged to avoid the negative consequences of an increased sedentary lifestyle [54].

## Overweight and obesity

Overweight/obesity is associated with arterial hypertension, diabetes mellitus, dyslipidaemia, inflammation, endothelial dysfunction, hypercoagulability and cardiac arrhythmia, which all increase vascular risk. Furthermore, overweight and obesity can restrict ventilation, diminish diaphragm excursion, forced respiratory volume and forced vital capacity and impair immune response to viral infections [3].

The COVID-19 pandemic increases risk of overweight/obesity, by social distancing, lockdown policies, economic downturn, decreased physical activity and modifications of lifestyle [32, 33].

A cohort study (*n* = 100 individuals) found an increased weight after 49 days of lockdown in 40% of individuals [55].

A case-control study (*n* = 32,583, 12,304 patients with newly diagnosed COVID-19) identified obesity to be a major risk factor for COVID-19 (adjusted OR 6.92, 95% CI 5.54–8.65; *p* < 0.0001) [56].

There is evidence that obesity/overweight increases the risk of severe COVID-19. A descriptive multicentre study (*n* = 1687) found that patients who were overweight (BMI 25–29.9 kg/m^2^, OR 1.32, 95% CI 1.03–1.69; *p* = 0.05), or had mild-to-moderate obesity (BMI = 30–39.9 kg/m^2^, OR 1.8, 95% CI 1.39–2.35; *p* = 0.05), or had morbid obesity (BMI > 40 kg/m^2^, OR 1.74, 95% CI 1.08–2.8; *p* = 0.05) were likelier to have severe COVID-19 vs. patients with normal weight [57]. Other studies found similar results [2, 58, 59].

Also, obesity has been shown to shift severe COVID-19 to younger ages. A review of a dataset of 265 hospitalized COVID-19 patients found a significant negative association between BMI and age (*p* = 0.0002). The median BMI was 29.3 kg/m^2^. Twenty-five percent of individuals had a BMI < 26 kg/m^2^ and 25% a BMI > 34.7 kg/m^2^ [4].

Potential explanations for these results might be the findings mentioned earlier in this review linked to obesity and overweight, such as mechanical restriction of ventilation and impaired immune response but also the higher expression of ACE2 in adipose tissue [60].

Overall, obese/overweight people are a special risk group of patients in light of COVID-19 and are at risk of having more severe COVID-19 [2].

## Dyslipidaemia

A retrospective study (*n* = 597, of whom 171 had severe and 32 critical COVID-19) observed a drop in high- (HDL-C) and low-density-lipoprotein cholesterol (LDL-C) during the COVID-19 course [61]. The latter finding was associated with worse outcome. Possible explanations for the drop in cholesterol include impaired lipid biogenesis because of liver injury, inflammatory-mediated modulation of lipid metabolism facilitating cholesterol clearance and increased vascular permeability due to infection and leakage of cholesterol molecules into the tissue [61].

In addition to lifestyle modifications, drugs are recommended to lower serum-cholesterol levels (https://www.who.int/).

Statins are widely used in patients with vascular disease on the basis of their lipid-lowering activity but also for their pleiotropic effects. They have an antiinflammatory effect, which is mainly mediated through pathways that lead to inhibition of nuclear factor kappa (NF-kB) [62]. Like ACEIs/ARBs, statins upregulate tissue ACE2 [63]. The CORONADO study did not find a protective role of statins on the primary endpoint of endotracheal intubation or death in COVID-19 patients with diabetes mellitus [58•]. Moreover, statins have been shown to increase interleukin-18, which may facilitate severe COVID-19 [63]. However, in a retrospective study (*n* = 13,981 COVID-19 patients, 1219 received statins), compared with individuals without pre-existing statin intake, individuals with in-hospital statin intake had a lower crude 28-day mortality (RR 0.78, 95% CI 0.61–0.996; *p* = 0.046) [64•]. Also, statins were shown to have a protective effect against SARS-CoV2 in silico [65].

## Smoking

Smoking probably has increased during the COVID-19 pandemic due to stress/depression, anxiety and fewer possibilities of outdoor physical and other activities. Also, face-to-face interventions aimed at supporting those who quit smoking have been made difficult [66]. However, a single-centre descriptive study (*n* = 357) found a higher rate of smoking cessation during the COVID-19 pandemic compared with 2019 (31.1% vs. 23.7%) [67].

Interestingly, upregulation of tissue ACE2 in smokers has been demonstrated [68].

A prospective cohort study (*n* = 387,109, 760 with COVID-19) found smoking to be a risk factor for COVID-19 (OR 1.42, 95% CI 1.12–1.79) [53•].

Smoking has been shown to impact outcome in COVID-19. A meta-analysis including a large number of COVID-19 patients (15 studies, *n* = 2473, 221 current and ex-smokers) showed that current smokers were more likely to have severe COVID-19 compared with former and never smokers (RR 1.45, 95% CI = 1.03–2.04) [69]. One study (*n* = 78 COVID-19 patients) identified smoking to be a strong risk factor for COVID-19 disease progression (OR 8.772, 95% CI = 1.942–40.000; *p* = 0.016). Disease progression was defined as change from mild/moderate to severe or critical disease or mortality and as severe to critical disease or mortality [8]. A meta-analysis (*n* = 6515) found current vs. non-current smoking to be a risk factor for adverse outcome (OR 1.53, 95% CI 1.06–2.2; *p* = 0.022) among hospitalized COVID-19 patients [70]. Contradicting the above results, a meta-analysis (*n* = 5960) showed a lower pooled prevalence of current smoking in hospitalized COVID-19 patients (6.5%, 95% CI 4.9–8.2%) compared with the prevalence of smoking in the general population [71].

In conclusion, cessation of smoking reduces the risk of stroke and according to most data—at least indirectly—also the burden of COVID-19.

## Unhealthy diet and overconsumption of alcohol

Dietary habits have changed during the COVID-19 pandemic. Social distancing, lockdown and economic downturn have impacted grocery shopping. People started favouring long-lasting and less healthy foods. As some people started experiencing more mental health problems than normally, it is likely that some increased their intake of alcohol [71]. One analysis showed a growth of alcohol online sales of 50–500% compared with the same period in 2019 [72]. However, other people have decreased their alcohol consumption, since there suddenly were less social events and restricted access to alcohol [73]. An electronic survey (*n* = 1047) showed alcohol binge drinking having decreased significantly during lockdown. Authors also reported that meal patterns during lockdown (type of food, eating out of control, intake of snacks between meals, number of main meals) were unhealthier [55]. However, a survey study from Italy (*n* = 3533) found an increased adherence to Mediterranean diet in the group aged 18–30 years during lockdown, compared with the younger and older populations (*p* < 0.001; *p* < 0.001, respectively) [74].

Ethanol suppresses the function of the immune system [75]. A meta-analysis (*n* = 1998) did not find an association between alcohol consumption and severity of COVID-19 (RR 1.09, 95% CI = 0.79–1.49; *p* = 0.6). However, the study did not consider the amount and duration of drinking [76].

## Cardiovascular disease

As mentioned earlier in this review, acute COVID-19 cardiovascular syndrome (ACovCS) has been described, potentially increasing the risk of stroke [12]. As the INTERSTROKE study has shown for stroke, the INTERHEART study has shown for cardiovascular events that with best management of the most frequent modifiable vascular risk factors, the vast majority of cardiovascular events could be prevented [77].

## Diabetes mellitus

Lockdown has been shown to increase the risk of diabetes mellitus [55, 78]. A cohort study (*n* = 100 non-diabetic individuals, follow-up 49 days) identified an increased risk of new diabetes mellitus of 7% [55]. A prospective cohort study (*n* = 387,109 individuals, 760 with COVID-19) found more hospitalized COVID-19 patients to have diabetes mellitus than not (9.5% vs. 4.8%) [53•].

A meta-analysis (*n* = 78,874) showed that diabetes mellitus was associated with severe COVID-19 (OR 2.10, 95% CI = 1.71–2.56; *p* < 0.001) and mortality (OR 2.78, 95% CI = 2.09–3.44) [6]. Also, in another meta-analysis (*n* = 1576, 280 with severe COVID-19), diabetes mellitus was the most frequent vascular risk factor (9.7%, 95% CI = 7.2–12.2%), after arterial hypertension [28].

Furthermore, a retrospective multicentre study (*n* = 7337 patients with COVID-19, 952 with pre-existing diabetes mellitus) found that in-hospital well-controlled blood glucose (glycaemic variability 3.9–10 mmol/l) was associated with significantly lower mortality compared with patients with poor glycaemic control (OR 0.13, 95% CI 0.04–0.44; *p* < 0.001) [79••].

Several potential mechanisms increasing this risk of COVID-19 in patients with diabetes mellitus have been proposed: (1) higher affinity of cellular binding of SARS-CoV-2 and higher levels of circulating furin facilitating virus entry, (2) increased ACE2 expression in the lungs, (3) decreased viral clearance, (4) diminished T cell function, (5) increased susceptibility to inflammation and cytokine storm syndrome and (6) co-existence of vascular disease and risk factors [5].

Of note, the CORONADO study, a multicentre observational study in hospitalized COVID-19 patients with diabetes mellitus (*n* = 1,317,410 admitted to ICU), did not find an association in univariate analysis between the primary endpoint (tracheal intubation and/or death within 7 days of admission) and age, type of diabetes mellitus, HbA1c levels, diabetic complications, or antidiabetic therapy [58•]. Noteworthy, in this study, the glycaemic control was not measured by serum glucose, potentially helping to explain the findings contradicting other studies.

In conclusion, diabetes mellitus not only increases the risk of stroke but also increased the burden of COVID-19 in most studies.

Antidiabetic drugs such as glucagon-like peptide-1 agonists, inhibitors of sodium-glucose transporter 2 and thiazolidinediones are known to increase the expression of ACE2 [80].

A retrospective study (*n* = 403 patients with COVID-19, 85 of whom with type 2 diabetes) found no significant effect of intake of dipeptidyl peptidase-4 on outcome [81]. Another retrospective analysis (*n* = 283 patients with COVID-19 and diabetes type 2) identified lower mortality rates in patients with vs. without metformin intake (*p* = 0.01) [82]. Further high-quality data regarding outcome of COVID-19 in patients with intake of antidiabetic drugs are yet lacking [83].

## Mental health problems

Lockdown, social distancing, economic downturn, rapid transmission of infection, high number of deaths and lack of effective treatment strategies and vaccines may facilitate mental health problems not only in patients with pre-existing mental disease but also in healthy persons, causing fear, anxiety, depression, stress reactions, posttraumatic stress disorder and sleep problems [32, 84].

An online survey (*n* = 1210) analysed mental health impact of the COVID-19 outbreak. Responders (53.8%, significantly more female) reported moderate to severe avoidance, intrusion and/or hyperarousal, 16.5% (significantly more male) moderate to severe depressive symptoms and 28.8% (significantly more male) moderate to severe/extremely severe anxiety [32]. Being a student or reporting poor/very poor self-rated health status was significantly associated with higher mental health impact compared with not being a student or compared with those with good/very good self-rated health status. Uneducated status was significantly associated with depressive symptoms [32].

An internet-based, cross-sectional survey (*n* = 1160) showed higher rates of depression (assessed using Epidemiological Studies Depression Scale) and anxiety in quarantined vs. non-quarantined responders (36.05% vs. 16.12%) [85]. However, another survey study (*n* = 1443 participants in quarantine, 836 without quarantine) did not find an impact of quarantine status on depression likelihood (using Patient Health Questionnaire-9) [86].

A cross-sectional study (*n* = 932) found female sex, young age, low annual income, current smoking and physical multimorbidity to be associated with mental health issues during the COVID-19 pandemic [84].

Mental disease has been identified to be a risk factor for infection with COVID-19. Possible explanations are cognitive impairment, little awareness of risk and diminished efforts regarding personal protection as well as confined conditions in psychiatric wards, which make social distancing and compliance with hygiene measures more difficult. Also, access to timely health care may be difficult for patients with mental disease due to the stigma [87].

A cross-sectional study (*n* = 3947) found a higher likelihood of depression (assessed using Patient Health Questionnaire-9) in people with suspected COVID-19 symptoms (OR = 2.88, 95% CI 2.18–3.8; *p* < 0.001). An online survey study (*n* = 770 COVID-19 patients) found a prevalence of depression (using PHQ-9) of 43.1% in clinically stable patients [88].

One prospective cross-sectional study (*n* = 114 COVID-19 patients) found a significant association of loss of smell and depression and anxiety (assessed using PHQ-2 and generalized anxiety disorder-2) [89].

To reduce the psychological impact of the COVID-19 pandemic, the WHO published simple recommendations: getting up and going to bed at similar times every day, keeping up with personal hygiene, eating healthy meals at regular times, exercising regularly, allocating time for working and time for resting, making time for enjoyable things, minimizing newsfeeds, limiting the amounts of alcohol and drug consumption and limiting screen time (https://www.who.int/).

Strategies to reduce the burden of mental disease related to COVID-19 have been proposed such as development of teams of specialists qualified to address emotional distress, training of community health personnel in basic aspects of mental health care, use of online surveys to assess the scope of mental health problems, development of online materials for mental health education, provision of online counselling and self-help services, use of structured letters as a form of asynchronous telepsychiatry consultation and development of synchronous telemedicine services for diagnostic purposes as well as counselling. Also, there is a need to make online mental health services accessible to individuals from lower socioeconomic background [89].

## Conclusion

The relationship between vascular events, vascular disease and vascular risk factors—and COVID-19—is strongly intertwined. There is a synergism between what is healthy and what makes us less vulnerable to contract COVID-19 and to what increases the risk of having severe COVID-19 and bad outcome. Not only optimal secondary but also primary vascular prevention might reduce the burden of COVID-19 in our ageing populations.

## Data Availability

Data sharing is not applicable to this article as no new data were created or analysed in this review.
